# Poly[[triaqua(butane-1,2,3,4-tetra­carboxyl­ato)dimanganese(II)] mono­hydrate]

**DOI:** 10.1107/S1600536809042998

**Published:** 2009-10-23

**Authors:** Ling Wu

**Affiliations:** a Jilin Agriculture Engineering Polytechnic College, Siping 136000, People’s Republic of China

## Abstract

The asymmetric unit of the title Mn^II^ coordination polymer, {[Mn_2_(C_8_H_6_O_8_)(H_2_O)_3_]·H_2_O}_*n*_, contains two crystallographic­ally independent Mn^II^ cations, two half butane-1,2,3,4-tetra­carboxyl­ato anions, each lying on a centre of inversion, and four water mol­ecules. The Mn^II^ cation has a distorted octa­hedral coordination environment. One Mn centre is coordinated by four carboxyl­ate O atoms from two different anions and two water O atoms. The other Mn centre is coordinated by five carboxyl­ate O atoms from four different anions and one water O atom. One water mol­ecule does not coordinate to a Mn centre. The crystal packing is stabilized by several O—H⋯O hydrogen bonds, forming a three-dimensional framework.

## Related literature

For multicarboxyl­ate ligands in the construction of coordin­ation polymers, see: Yang *et al.* (2008 ▶). For butane-1,2,3,4-tetracarboxylic acid in coordination chemistry, see: Liu *et al.* (2008 ▶).
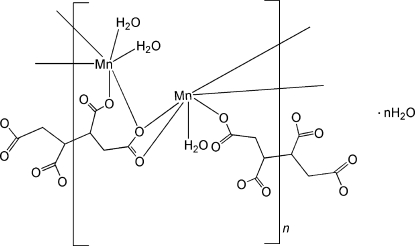


## Experimental

### 

#### Crystal data

[Mn_2_(C_8_H_6_O_8_)(H_2_O)_3_]·H_2_O
*M* *_r_* = 824.14
Monoclinic, 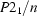

*a* = 8.1962 (4) Å
*b* = 12.3291 (7) Å
*c* = 12.9758 (6) Å
β = 97.760 (5)°
*V* = 1299.22 (11) Å^3^
*Z* = 2
Mo *K*α radiation
μ = 2.01 mm^−1^
*T* = 293 K
0.33 × 0.21 × 0.17 mm

#### Data collection

Bruker APEX CCD area-detector diffractometer
Absorption correction: multi-scan (*SADABS*; Sheldrick, 1996 ▶) *T*_min_ = 0.764, *T*_max_ = 0.852
7085 measured reflections
3031 independent reflections
1990 reflections with *I* > 2σ(*I*)
*R*_int_ = 0.040

#### Refinement

*R*[*F* ^2^ > 2σ(*F* ^2^)] = 0.041
*wR*(*F* ^2^) = 0.106
*S* = 0.88
3031 reflections
227 parameters
12 restraints
H atoms treated by a mixture of independent and constrained refinement
Δρ_max_ = 0.63 e Å^−3^
Δρ_min_ = −0.83 e Å^−3^

### 

Data collection: *SMART* (Bruker, 1997 ▶); cell refinement: *SAINT* (Bruker, 1999 ▶); data reduction: *SAINT*; program(s) used to solve structure: *SHELXS97* (Sheldrick, 2008 ▶); program(s) used to refine structure: *SHELXL97* (Sheldrick, 2008 ▶); molecular graphics: *XP* (Sheldrick, 2008 ▶); software used to prepare material for publication: *SHELXL97*.

## Supplementary Material

Crystal structure: contains datablocks global, I. DOI: 10.1107/S1600536809042998/bt5105sup1.cif

Structure factors: contains datablocks I. DOI: 10.1107/S1600536809042998/bt5105Isup2.hkl

Additional supplementary materials:  crystallographic information; 3D view; checkCIF report

## Figures and Tables

**Table 1:** Hydrogen-bond geometry (Å, °)

*D*—H⋯*A*	*D*—H	H⋯*A*	*D*⋯*A*	*D*—H⋯*A*
O2*W*—H*W*22⋯O6^i^	0.843 (10)	2.46 (5)	2.998 (4)	122 (4)
O2*W*—H*W*22⋯O4*W*	0.843 (10)	2.39 (4)	2.985 (10)	128 (4)
O3*W*—H*W*31⋯O4^ii^	0.850 (10)	2.075 (19)	2.874 (4)	156 (4)
O4*W*—H*W*41⋯O1^ii^	0.86 (12)	2.43 (14)	3.091 (12)	133 (16)
O4*W*—H*W*42⋯O6^iii^	0.85 (13)	2.58 (14)	3.124 (11)	123 (14)
O1*W*—H*W*11⋯O7^iv^	0.850 (10)	2.098 (11)	2.944 (4)	173 (4)
O1*W*—H*W*12⋯O3^ii^	0.854 (10)	2.39 (4)	2.935 (4)	123 (3)
O2*W*—H*W*21⋯O2^iii^	0.845 (10)	1.919 (18)	2.731 (4)	161 (4)
O3*W*—H*W*32⋯O2*W*^v^	0.849 (10)	2.333 (17)	3.155 (5)	163 (5)

Symmetry codes: (i) 
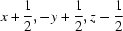
; (ii) 
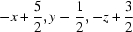
; (iii) 
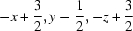
; (iv) 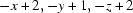; (v) 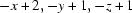.

**Fig. 1.**
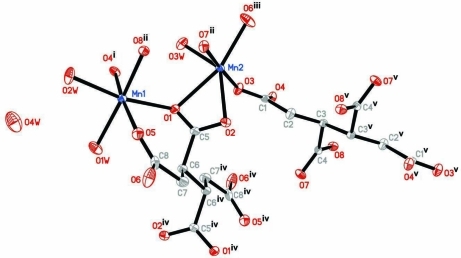
A view of the local coordination of the MnII cations in (I), showing the atom-numbering scheme. Displacement ellipsoids at the 30% probability level (symmetry operations i: 2.5-x, y-0.5, 1.5-z; ii: x-0.5, 1.5-y, z-0.5; iii: 1.5-x, y+0.5, 1.5-z; iv: 2-x, 1-y, 2-z); v: 2-x, 2-y, 2-z).

## supplementary crystallographic information

### Comment

So far, the multicarboxylate ligands, such as 1,2,3-benzenedicarboxylic acid, 1,2,4-benzenedicarboxylic acid and 1,3,5-benzenedicarboxylic acid, are widely used to construct the coordination polymers with interesting properties (Yang *et al.* 2008). In this regard, butane-1,2,3,4-tetracarboxylatic acid (H_4_L) is also a good ligand in coordination chemistry due to its strong coordination ability and versatile coordination modes, so much attention has been paid to it in recent years (Liu *et al.* 2008). In this contribution, H_4_L was selected as a bridging ligand, and a new manganese coordination polymer, namely [Mn_2_(*L*)(H_2_O)_3_]^.^H_2_O (I).

As shown in Fig. 1, the asymmetric unit of (I) contains two crystallographically Mn^II^ cation, two half *L* anions and four water molecules. The *L* ligand ligand is at an inversion center. Each Mn^II^ cation has a distorted octahedral coordination environment. Mn1 is coordinated by four carboxylate O atoms from two different *L* anions and two water O atoms. Mn2 is coordinated by five carboxylate O atoms from three different *L* anions and one water O atom. The *L* ligands bridging the neighboring Mn^II^ centers to form a complicated three-dimensional framework structure of (I) (Fig. 2). The hydrogen-bonding interactions between the water molecules and the carboxylate O atoms further stabilize the three-dimensional framework structure of (I).

### Experimental

A mixture of Mn(NO_3_)_2_.6H_2_O (0.10 mmol), H_4_L (0.05 mmol) and water (12 ml) was sealed in a Teflon reactor (15 ml), which was heated at 140 °C for 3 days and then gradually cooled to room temperature. Purple crystals of (I) were isolated (yield 64% based on Mn).

### Refinement

H atoms bonded to C atom were positioned geometrically (C—H = 0.93 Å) and refined as riding, with *U*_iso_(H)=1.2*U*_eq_(carrier). The water H-atoms were located in a difference Fourier map, and were refined with distance restraints of O–H = 0.85±0.01 Å and H···H = 1.35±0.01 Å; their temperature factors were tied to those of parent atoms by a factor of 1.5.

### Crystal data

**Table:**

[Mn_2_(C_8_H_6_O_8_)(H_2_O)_3_]·H_2_O	*F*(000) = 832
*M**_r_* = 824.14	*D*_x_ = 2.107 Mg m^−^^3^
Monoclinic, *P*2_1_/*n*	Mo *K*α radiation, λ = 0.71073 Å
Hall symbol: -P 2yn	Cell parameters from 3031 reflections
*a* = 8.1962 (4) Å	θ = 3.0–29.1°
*b* = 12.3291 (7) Å	µ = 2.01 mm^−^^1^
*c* = 12.9758 (6) Å	*T* = 293 K
β = 97.760 (5)°	Block, colorless
*V* = 1299.22 (11) Å^3^	0.33 × 0.21 × 0.17 mm
*Z* = 2	

### Data collection

**Table:**

Bruker APEX CCD area-detector diffractometer	3031 independent reflections
Radiation source: fine-focus sealed tube	1990 reflections with *I* > 2σ(*I*)
graphite	*R*_int_ = 0.040
ω scans	θ_max_ = 29.1°, θ_min_ = 3.0°
Absorption correction: multi-scan (*SADABS*; Sheldrick, 1996)	*h* = −11→7
*T*_min_ = 0.764, *T*_max_ = 0.852	*k* = −12→15
7085 measured reflections	*l* = −17→17

### Refinement

**Table:**

Refinement on *F*^2^	Primary atom site location: structure-invariant direct methods
Least-squares matrix: full	Secondary atom site location: difference Fourier map
*R*[*F*^2^ > 2σ(*F*^2^)] = 0.041	Hydrogen site location: inferred from neighbouring sites
*wR*(*F*^2^) = 0.106	H atoms treated by a mixture of independent and constrained refinement
*S* = 0.88	*w* = 1/[σ^2^(*F*_o_^2^) + (0.0651*P*)^2^] where *P* = (*F*_o_^2^ + 2*F*_c_^2^)/3
3031 reflections	(Δ/σ)_max_ < 0.001
227 parameters	Δρ_max_ = 0.63 e Å^−^^3^
12 restraints	Δρ_min_ = −0.83 e Å^−^^3^

### Special details

**Table:**

Geometry. All e.s.d.'s (except the e.s.d. in the dihedral angle between two l.s. planes) are estimated using the full covariance matrix. The cell e.s.d.'s are taken into account individually in the estimation of e.s.d.'s in distances, angles and torsion angles; correlations between e.s.d.'s in cell parameters are only used when they are defined by crystal symmetry. An approximate (isotropic) treatment of cell e.s.d.'s is used for estimating e.s.d.'s involving l.s. planes.
Refinement. Refinement of *F*^2^ against ALL reflections. The weighted *R*-factor *wR* and goodness of fit *S* are based on *F*^2^, conventional *R*-factors *R* are based on *F*, with *F* set to zero for negative *F*^2^. The threshold expression of *F*^2^ > σ(*F*^2^) is used only for calculating *R*-factors(gt) *etc*. and is not relevant to the choice of reflections for refinement. *R*-factors based on *F*^2^ are statistically about twice as large as those based on *F*, and *R*- factors based on ALL data will be even larger.

### Fractional atomic coordinates and isotropic or equivalent isotropic displacement parameters (Å^2^)

**Table:**

	*x*	*y*	*z*	*U*_iso_*/*U*_eq_	
C1	1.1879 (4)	0.8429 (3)	0.8558 (3)	0.0198 (8)	
C2	1.0480 (4)	0.8783 (3)	0.9135 (3)	0.0271 (9)	
H2A	1.0241	0.8197	0.9590	0.033*	
H2B	0.9507	0.8893	0.8630	0.033*	
C3	1.0781 (4)	0.9804 (3)	0.9778 (3)	0.0205 (7)	
H3	1.1109	1.0378	0.9325	0.025*	
C4	1.2178 (4)	0.9664 (3)	1.0691 (3)	0.0193 (8)	
C5	0.9481 (4)	0.5441 (3)	0.8594 (3)	0.0193 (8)	
C6	0.9556 (4)	0.4695 (3)	0.9522 (2)	0.0220 (8)	
H6	1.0203	0.4052	0.9395	0.026*	
C7	0.7837 (5)	0.4336 (3)	0.9692 (3)	0.0306 (9)	
H7A	0.7915	0.3939	1.0343	0.037*	
H7B	0.7172	0.4976	0.9760	0.037*	
C8	0.6978 (5)	0.3639 (3)	0.8845 (3)	0.0275 (9)	
O1	1.0249 (3)	0.5178 (2)	0.78555 (18)	0.0248 (6)	
O2	0.8740 (3)	0.6329 (2)	0.85773 (19)	0.0263 (6)	
O1W	1.0570 (4)	0.2456 (3)	0.8215 (3)	0.0391 (8)	
O3	1.1646 (3)	0.7613 (2)	0.7983 (2)	0.0287 (6)	
O2W	0.9047 (4)	0.2290 (3)	0.5915 (3)	0.0425 (8)	
HW22	0.969 (4)	0.181 (3)	0.575 (4)	0.064*	
O4	1.3228 (3)	0.8944 (2)	0.87012 (19)	0.0253 (6)	
O3W	1.1264 (4)	0.6245 (3)	0.6096 (2)	0.0357 (7)	
HW31	1.129 (6)	0.5569 (10)	0.597 (3)	0.054*	
O5	0.7513 (3)	0.3496 (2)	0.7980 (2)	0.0317 (7)	
O4W	1.0955 (15)	0.0359 (7)	0.6785 (11)	0.192 (4)	
HW41	1.181 (13)	0.011 (12)	0.654 (14)	0.288*	
HW42	1.06 (2)	−0.021 (8)	0.705 (14)	0.288*	
O6	0.5696 (4)	0.3186 (3)	0.9039 (3)	0.0458 (9)	
O7	1.2229 (3)	0.8806 (2)	1.12238 (19)	0.0268 (6)	
O8	1.3177 (3)	1.0437 (2)	1.08580 (19)	0.0253 (6)	
Mn1	0.96260 (7)	0.36380 (5)	0.70273 (4)	0.02057 (16)	
Mn2	0.95382 (6)	0.68692 (4)	0.70684 (4)	0.01775 (15)	
HW11	0.979 (4)	0.205 (3)	0.835 (4)	0.064 (18)*	
HW12	1.129 (4)	0.203 (3)	0.802 (4)	0.065 (18)*	
HW21	0.812 (2)	0.198 (3)	0.592 (4)	0.041 (14)*	
HW32	1.102 (6)	0.654 (3)	0.5505 (18)	0.063 (18)*	

### Atomic displacement parameters (Å^2^)

**Table:**

	*U*^11^	*U*^22^	*U*^33^	*U*^12^	*U*^13^	*U*^23^
C1	0.0179 (17)	0.025 (2)	0.0152 (16)	0.0031 (16)	−0.0039 (14)	0.0026 (15)
C2	0.0181 (18)	0.032 (2)	0.0288 (19)	0.0020 (17)	−0.0043 (16)	−0.0103 (17)
C3	0.0188 (17)	0.0238 (19)	0.0173 (16)	0.0020 (16)	−0.0032 (14)	−0.0006 (15)
C4	0.0162 (16)	0.023 (2)	0.0180 (16)	0.0050 (16)	−0.0016 (14)	−0.0002 (16)
C5	0.0191 (17)	0.021 (2)	0.0153 (16)	−0.0061 (16)	−0.0080 (14)	−0.0003 (14)
C6	0.0279 (18)	0.0190 (19)	0.0169 (16)	−0.0047 (17)	−0.0054 (15)	−0.0017 (15)
C7	0.035 (2)	0.030 (2)	0.0261 (19)	−0.010 (2)	−0.0026 (17)	0.0011 (18)
C8	0.026 (2)	0.017 (2)	0.036 (2)	0.0032 (17)	−0.0059 (18)	−0.0048 (17)
O1	0.0317 (14)	0.0231 (14)	0.0188 (12)	−0.0017 (12)	0.0005 (11)	−0.0016 (11)
O2	0.0284 (14)	0.0264 (16)	0.0232 (13)	0.0014 (12)	0.0005 (11)	0.0024 (12)
O1W	0.0288 (16)	0.0375 (19)	0.0508 (19)	0.0121 (16)	0.0045 (15)	0.0166 (16)
O3	0.0197 (13)	0.0327 (16)	0.0328 (14)	−0.0037 (12)	0.0006 (11)	−0.0140 (13)
O2W	0.0367 (17)	0.0322 (18)	0.061 (2)	−0.0119 (15)	0.0148 (16)	−0.0229 (16)
O4	0.0191 (12)	0.0246 (15)	0.0323 (14)	−0.0045 (11)	0.0040 (11)	−0.0093 (12)
O3W	0.0359 (17)	0.0418 (19)	0.0312 (16)	−0.0032 (15)	0.0116 (14)	−0.0101 (14)
O5	0.0291 (15)	0.0325 (17)	0.0296 (15)	0.0015 (13)	−0.0098 (12)	−0.0031 (13)
O4W	0.179 (9)	0.162 (8)	0.243 (11)	0.014 (6)	0.060 (8)	0.014 (8)
O6	0.0260 (15)	0.047 (2)	0.065 (2)	−0.0131 (15)	0.0074 (15)	−0.0301 (17)
O7	0.0205 (12)	0.0259 (16)	0.0313 (14)	0.0006 (11)	−0.0070 (11)	0.0080 (12)
O8	0.0227 (13)	0.0288 (16)	0.0226 (12)	−0.0054 (12)	−0.0035 (10)	0.0006 (11)
Mn1	0.0198 (3)	0.0200 (3)	0.0207 (3)	−0.0005 (2)	−0.0016 (2)	0.0007 (2)
Mn2	0.0165 (3)	0.0188 (3)	0.0166 (3)	0.0002 (2)	−0.0029 (2)	0.0014 (2)

### Geometric parameters (Å, °)

**Table:**

C1—O3	1.252 (4)	O1—Mn2	2.360 (3)
C1—O4	1.267 (4)	O2—Mn2	2.247 (2)
C1—C2	1.515 (5)	O1W—Mn1	2.185 (3)
C2—C3	1.512 (5)	O1W—HW11	0.850 (10)
C2—H2A	0.9700	O1W—HW12	0.854 (10)
C2—H2B	0.9700	O3—Mn2	2.163 (3)
C3—C4	1.542 (5)	O2W—Mn1	2.211 (3)
C3—C3^i^	1.549 (7)	O2W—HW22	0.843 (10)
C3—H3	0.9800	O2W—HW21	0.845 (10)
C4—O8	1.257 (4)	O4—Mn1^iii^	2.140 (2)
C4—O7	1.262 (4)	O3W—Mn2	2.160 (3)
C5—O2	1.251 (4)	O3W—HW31	0.850 (10)
C5—O1	1.258 (4)	O3W—HW32	0.849 (10)
C5—C6	1.510 (5)	O5—Mn1	2.267 (3)
C6—C7	1.521 (5)	O4W—HW41	0.86 (12)
C6—C6^ii^	1.546 (7)	O4W—HW42	0.85 (13)
C6—H6	0.9800	O6—Mn2^iv^	2.160 (3)
C7—C8	1.494 (6)	O7—Mn2^v^	2.217 (3)
C7—H7A	0.9700	O8—Mn1^v^	2.127 (3)
C7—H7B	0.9700	Mn1—O8^vi^	2.127 (3)
C8—O6	1.245 (5)	Mn1—O4^vii^	2.140 (2)
C8—O5	1.271 (5)	Mn2—O6^viii^	2.160 (3)
O1—Mn1	2.207 (3)	Mn2—O7^vi^	2.217 (3)
			
O3—C1—O4	123.3 (3)	C1—O3—Mn2	135.5 (2)
O3—C1—C2	117.5 (3)	Mn1—O2W—HW22	128 (3)
O4—C1—C2	119.2 (3)	Mn1—O2W—HW21	116 (3)
C1—C2—C3	115.7 (3)	HW22—O2W—HW21	106.4 (17)
C1—C2—H2A	108.3	C1—O4—Mn1^iii^	126.9 (2)
C3—C2—H2A	108.3	Mn2—O3W—HW31	120 (3)
C1—C2—H2B	108.3	Mn2—O3W—HW32	106 (3)
C3—C2—H2B	108.3	HW31—O3W—HW32	104.9 (16)
H2A—C2—H2B	107.4	C8—O5—Mn1	148.3 (3)
C2—C3—C4	112.3 (3)	HW41—O4W—HW42	101 (13)
C2—C3—C3^i^	112.5 (4)	C8—O6—Mn2^iv^	101.8 (2)
C4—C3—C3^i^	108.3 (3)	C4—O7—Mn2^v^	123.3 (2)
C2—C3—H3	107.8	C4—O8—Mn1^v^	144.9 (2)
C4—C3—H3	107.8	O8^vi^—Mn1—O4^vii^	90.20 (9)
C3^i^—C3—H3	107.8	O8^vi^—Mn1—O1W	166.12 (11)
O8—C4—O7	124.7 (3)	O4^vii^—Mn1—O1W	101.26 (10)
O8—C4—C3	116.5 (3)	O8^vi^—Mn1—O1	87.51 (10)
O7—C4—C3	118.8 (3)	O4^vii^—Mn1—O1	85.02 (10)
O2—C5—O1	120.3 (3)	O1W—Mn1—O1	101.15 (11)
O2—C5—C6	120.9 (3)	O8^vi^—Mn1—O2W	83.53 (12)
O1—C5—C6	118.7 (3)	O4^vii^—Mn1—O2W	87.77 (11)
C5—C6—C7	110.8 (3)	O1W—Mn1—O2W	89.06 (14)
C5—C6—C6^ii^	107.9 (4)	O1—Mn1—O2W	168.48 (11)
C7—C6—C6^ii^	111.8 (4)	O8^vi^—Mn1—O5	92.08 (10)
C5—C6—H6	108.8	O4^vii^—Mn1—O5	171.42 (10)
C7—C6—H6	108.8	O1W—Mn1—O5	77.75 (11)
C6^ii^—C6—H6	108.8	O1—Mn1—O5	86.81 (9)
C8—C7—C6	114.5 (3)	O2W—Mn1—O5	100.70 (11)
C8—C7—H7A	108.6	O3W—Mn2—O6^viii^	83.43 (13)
C6—C7—H7A	108.6	O3W—Mn2—O3	86.20 (11)
C8—C7—H7B	108.6	O6^viii^—Mn2—O3	92.23 (12)
C6—C7—H7B	108.6	O3W—Mn2—O7^vi^	99.22 (11)
H7A—C7—H7B	107.6	O6^viii^—Mn2—O7^vi^	87.70 (12)
O6—C8—O5	121.2 (4)	O3—Mn2—O7^vi^	174.53 (10)
O6—C8—C7	115.8 (3)	O3W—Mn2—O2	133.95 (12)
O5—C8—C7	123.0 (4)	O6^viii^—Mn2—O2	142.39 (11)
C5—O1—Mn1	119.0 (2)	O3—Mn2—O2	87.30 (10)
C5—O1—Mn2	89.0 (2)	O7^vi^—Mn2—O2	89.42 (10)
Mn1—O1—Mn2	121.45 (11)	O3W—Mn2—O1	78.19 (11)
C5—O2—Mn2	94.4 (2)	O6^viii^—Mn2—O1	161.24 (11)
Mn1—O1W—HW11	110 (3)	O3—Mn2—O1	90.28 (10)
Mn1—O1W—HW12	114 (4)	O7^vi^—Mn2—O1	91.54 (10)
HW11—O1W—HW12	104.7 (16)	O2—Mn2—O1	56.30 (9)

Symmetry codes: (i) −*x*+2, −*y*+2, −*z*+2; (ii) −*x*+2, −*y*+1, −*z*+2; (iii) −*x*+5/2, *y*+1/2, −*z*+3/2; (iv) −*x*+3/2, *y*−1/2, −*z*+3/2; (v) *x*+1/2, −*y*+3/2, *z*+1/2; (vi) *x*−1/2, −*y*+3/2, *z*−1/2; (vii) −*x*+5/2, *y*−1/2, −*z*+3/2; (viii) −*x*+3/2, *y*+1/2, −*z*+3/2.

### Hydrogen-bond geometry (Å, °)

**Table:**

*D*—H···*A*	*D*—H	H···*A*	*D*···*A*	*D*—H···*A*
O2W—HW22···O6^ix^	0.84 (1)	2.46 (5)	2.998 (4)	122 (4)
O2W—HW22···O4W	0.84 (1)	2.39 (4)	2.985 (10)	128 (4)
O3W—HW31···O4^vii^	0.85 (1)	2.08 (2)	2.874 (4)	156 (4)
O4W—HW41···O1^vii^	0.86 (12)	2.43 (14)	3.091 (12)	133 (16)
O4W—HW42···O6^iv^	0.85 (13)	2.58 (14)	3.124 (11)	123 (14)
O1W—HW11···O7^ii^	0.85 (1)	2.10 (1)	2.944 (4)	173 (4)
O1W—HW12···O3^vii^	0.85 (1)	2.39 (4)	2.935 (4)	123 (3)
O2W—HW21···O2^iv^	0.85 (1)	1.92 (2)	2.731 (4)	161 (4)
O3W—HW32···O2W^x^	0.85 (1)	2.33 (2)	3.155 (5)	163 (5)

Symmetry codes: (ix) *x*+1/2, −*y*+1/2, *z*−1/2; (vii) −*x*+5/2, *y*−1/2, −*z*+3/2; (iv) −*x*+3/2, *y*−1/2, −*z*+3/2; (ii) −*x*+2, −*y*+1, −*z*+2; (x) −*x*+2, −*y*+1, −*z*+1.
